# Influencing Factors of Nurses’ Practice during the Bedside Handover: A Qualitative Evidence Synthesis Protocol

**DOI:** 10.3390/jpm13020267

**Published:** 2023-01-31

**Authors:** Paulo Cruchinho, Gisela Teixeira, Pedro Lucas, Filomena Gaspar

**Affiliations:** Nursing Research, Innovation and Development Centre of Lisbon (CIDNUR), Nursing School of Lisbon, 1600-096 Lisbon, Portugal

**Keywords:** change management, management, nursing, organizational innovation, patient-centered care, patient handoff, patient safety, quality improvement, qualitative research, systematic review

## Abstract

Nursing Bedside Handover (NBH) is acknowledged as a nursing practice implemented at the patient’s bedside to improve communication safety during the shift change, but it is vulnerable due to inconsistent application among nurses. This synthesis of qualitative evidence aims to review and synthesize the perceptions and experiences of nurses regarding the factors that, in their perspective, influence NBH practice. We will follow the thematic synthesis methodology of Thomas and Harden and the Enhancing Transparency in Reporting the Synthesis of Qualitative Research (ENTREQ) Statement guidelines. A search will be conducted through the databases of MEDLINE, CINAHL, Web of Science, and Scopus, and we will follow the three-step search process to identify primary studies with qualitative or mixed-method research designs and projects of quality improvement. The screening and selection of the studies will be carried out by two independent reviewers. We will use the Preferred Reporting Items for Systematic Reviews and Meta-analyses (PRISMA) to report the screening, search, and selection of studies. To assess its methodological quality, two reviewers will independently use the CASM Tool. The extracted data will be reviewed, categorized, and summarized in tabular and narrative formats. The findings obtained will allow us to inform future research and change management led by nurse managers.

## 1. Introduction

Clinical handover is one of the most common communication practices among healthcare workers [1]. It is defined by the Australian Medical Association [2] as a temporary or permanent transfer of the professional responsibility for some or all aspects of care regarding one patient or group of patients to another individual or professional group. In nursing, the nursing handover differs according to organizations and nursing teams.

In 2007, the World Health Organization, in its report, “Communication During Patient Hand-Overs”, recommended healthcare workers adopt a set of strategies to increase handover safety, among which included patient participation [3]. Since then, several studies and experiences about the change to the Nursing Bedside Handover (NBH) have been reported [4,5,6,7,8,9,10,11,12]. This type of clinical handover is distinguished by the patient-centered [11,12] and family [13,14] approach, by the direct involvement of patients [6,15,16], care givers, and relatives [17,18,19], and by inciting nurses to ask questions [10,20] and to express their opinions or comments in the handover [21,22]. It is also characterized by the sharing of information with patients [23,24,25] and families [14,26], which may require nurses to make a conscious choice of the information to be shared [27] and to discuss [28,29] and plan the care for the next shift together with the patient and family [30,31,32]. It also includes monitoring the evolution of the patient’s condition [33,34] and performing safety checks on the patients’ medical devices [35,36].

Several studies have reported positive outcomes from implementing the change to NBH. For instance, in a study conducted by Bradley and Mott [37], which compared the period before and after implementing the NBH, it was found that the NBH had a significant effect on the reduction of some adverse events, namely, medication errors, falls, pressure ulcers, and other skin lesions. Regarding the events related to medication, the work of Kerr et al. [38], which involved the analysis of 754 medical records, also verified that after 12 months of NBH implementation, nurses increased from 83.3 to 95.4% the identification of patients with drug allergies and from 81.1 to 97.3% the administration of prescribed medication. In another study, Wong et al. [39], motivated by the need to decrease medication incidents due to inconsistencies in the transmission of information between nurses, got a 73.68% reduction in the incidence of medication errors, from nineteen incidents before implementation to only five after six months of implementation. Sand-Jecklin and Sherman [40] also found a 50% reduction in medication errors after six months of implementation.

However, it’s known that NBH has a variable nature, both from the nurses’ and patients’ perspectives [1]. Some studies have verified that NBH is performed differently than planned [41], is repetitive [42], is not always performed, or only involves introducing the patient and the nurse starting the duty [43]. This variability has been reported in relation to individual nurse practices, in relation to NBH duration, the method used, the place of performance, and the information transmitted and shared with patients [44]. In some studies, inconsistencies were reported after NBH implementation, both from nurses and patients [40,43], even after standardizing the NBH process using nurse facilitators during the change process [43] and interventions aimed at increasing patient and family participation during NBH [45]. In a study aiming to determine the existence of compliance differences in the application of a structured NBH protocol in 12 different services of a Belgian hospital, Malfait et al. [46] found a unilateral decision of nurses to not perform NBH in almost 30% of the observed cases. It was also found that in one third of the cases in which the NBH was performed, nurses did not actively involve the patients [9]. The occurrence of resistance to the utilization of NBH had already been identified in previous studies for two reasons: (1) the NBH led outgoing nurses to leave late from their shifts and the incoming nurses to be late for their work [47,48]; and (2) the possibility of breaching the confidentiality of patient information [48].

In recent years, the explanation attributed to nurses’ inconsistency has come to consider, in addition to nurses’ resistance, the possibility that nurses’ practices during NBH are individualized, flexible, and adaptive. For example, in a study involving the survey of what is known in the literature about NBH prior to its implementation, to avoid resistance from nurses, Schirm et al. [49] suggested the promotion of decision-making in relation to NBH rather than its mandatory use. Moreover, in a qualitative systematic review of patients’ experiences, McCloskey et al. [50] also exposed that nurses use adaptive practices to deal with the personal factors of patients, relatives, and environmental factors that affect NBH. Tobiano et al. [51], in a systematic review exploring how patient participation is promoted during NBH, also highlighted the importance of nurses using flexible approaches to deal with patient confidentiality and information sensibility, as well as the importance of tailoring the process to patients’ abilities, preferences, and expectations. Finally, with regard to the content of NBH, in a systematic literature analysis by Buus et al. [52], it was reported that nurses use a flexible approach regarding the clinical information and that they personalize and negotiate the clinical knowledge they transmit according to: (1) the context of the patients’ clinical situation and (2) the needs of the nurses coming to duty. Based on these supplementary explanations for nurses’ inconsistent practice during NBH, we formulated the following review question:

What are the factors perceived by nurses that influence inconsistency of practice during NBH?

To analyze the complexity of nurses’ practice, Kim’s Conceptual Framework of Nursing Practice will be adopted [53]. This author divides nursing practice into two processes that influence each other (the process of deliberation and the process of enactment), both composed of three common elements: patients, nurses, and care settings [53]. Therefore, we formulated three sub-questions:

(1) What factors are related to patients?;
(2) What factors are related to nurses?;
(3) Additionally, what factors are related to the care setting?

### The Rationale for the Qualitative Evidence Synthesis

We identified three qualitative systematic reviews, one of which focused on patients’ experiences [54], another on the experiences of patients and nurses [55], and the third on the experiences of patients, nurses, and patients’ relatives [50]. However, no further qualitative evidence review was directed solely at the experiences of nurses to explore the factors that, in their point of view, influence NBH practices. To address this gap in the evidence, this review is based on Kim’s concept of nursing practice [53], defined by the cognitive, behavioral, and social aspects of the professional actions performed by nurses in the fulfillment of their role in a given situation. Therefore, it includes the way a nurse thinks, how they make decisions, how knowledge is transformed, and how they perform certain actions [53], and interventions. To fill this gap in the evidence, we aim to conduct this review to inform future research, advance nursing practice, and provide nurse managers with systematized information on these factors for change management within the NBH scope. One of the essential characteristics of nurse managers throughout the change management process is the acquisition of macro perspectives on change [56]. Addressing the factors that influence nurses’ behavior in carrying out NBH is recommended in order to prevent nurses from reverting to a nursing handover “away from the patients” [29].

## 2. Materials and Methods

### 2.1. Aim

This synthesis of the qualitative evidence aims to review and synthesize nurses’ perceptions and experiences about the factors that, in their perspective, influence the practice of NBH.

### 2.2. Study Design

This type of synthesis constitutes an integration method, a comparison, or a synthesis of qualitative evidence that allows for the identification of themes or constructs [57] as well as future implications [58]. The concept of qualitative evidence synthesis in the scope of this review underpins the acknowledgement of the importance of mixed studies and questionnaires with free-text data, together with qualitative studies, to enrich the synthesis [59]. We will follow the methodology of thematic synthesis described by Thomas and Harden [60] and the Enhancing Transparency in Reporting the Synthesis of Qualitative Research (ENTREQ) Statement guidelines [61]. We chose the Thomas and Harden methodology once it was developed to address people’s perspectives and experiences in the review questions. We will also use the guidelines of Preferred Reporting Items for Systematic Reviews and Meta-analyses (PRISMA) [62] and PEOS (Population, Exposure, Outcomes, Study designs). This framework will allow researchers to determine appropriate terms for the inclusion and exclusion criteria of qualitative studies [63]. This protocol was registered with the registration number INPLASY2022111013 [64].

### 2.3. Eligibility Criteria

#### 2.3.1. Population

It will include studies that involve nurses as participants, isolated from or together with patients, patients’ relatives, and other healthcare workers. Consequently, those that include only patients, patients’ relatives, other healthcare workers, and/or nursing students will be excluded. For nurses, all professional qualifications will be considered, including those of registered nurses, licensed nurses, nursing assistants, and advanced practice nurses.

#### 2.3.2. Exposure

We will include all studies focused on the implementation of NBH. To handle the terminological inconsistency existent in the scientific literature to define the communication at the patient’s bedside during nursing handover, we will analyze all articles that use the following terms: “Nursing Bedside Handover”, ”Nursing Bedside Handoff”, “Bedside Handover”, “Bedside handoff”, Shift-to-shift Bedside Handover”, “Shift-to-shift Bedside Handoff”, “Bedside Nurse-to-Nurse Handover”, “Bedside Nurse-to-Nurse Handoff”, and “Nurse Bedside Shift Report”. When there is no conceptual definition of NBH, we will use the concept of safe communication as described by Schuster and Nykolyn [65]. This concept specifies the set of activities of nurses to collect and share information, clarify, and verify the accuracy of the interpretations made, acting collaboratively with patients, families, and other healthcare workers to achieve common goals related to the safety of care. According to Hannawa [66], to achieve these goals, it is necessary that nurses use communication skills to: (1) transmit, collect, and exchange sufficient information for a shared understanding; (2) transmit and interpret information correctly, using communication among themselves to validate the accuracy of the content of the messages communicated; (3) express and interpret verbal messages in an unambiguous way, using their interaction with others to reduce uncertainty and doubt; (4) frame their interaction within the local circumstances, such as time pressure or environmental noise, which can produce barriers to a shared understanding; and (5) respond verbally and nonverbally to expressed needs and expectations that maximize the likelihood of understanding. Finally, all clinical settings of hospital and community healthcare organizations where nurses have been exposed to NBH will be considered, including long-term care units, emergency rooms, intensive care units, palliative care units, operating rooms, and labor and delivery. Limits of geographical locations are not relevant for this study.

#### 2.3.3. Outcomes

Another factor of inclusion is the studies with information about nurses’ perceptions and experiences related to the reasons for inconsistent practices in NBH. We define inconsistent NBH practice as any deviation that makes it difficult or impossible to perform NBH at the patient’s bedside or his/her involvement in the communication of nursing handover.

#### 2.3.4. Study Designs

It will include all available articles with the following characteristics: (1) primary empirical studies with qualitative or mixed designs; (2) projects of quality improvement; and (3) studies published in English and in journals with peer review.

Articles with the following characteristics will be excluded: (1) secondary research studies, theses and dissertations, literature reviews, and editorial articles; (2) primary research studies with quantitative design; and (3) studies not published in journals, published in other languages, or published in journals without information on peer review. Gray literature will also be excluded as it may be difficult to retrieve and because studies are not peer reviewed.

The review targets the studies published up to 31 October 2022.

### 2.4. Search Strategy

The literature search will be conducted through the MEDLINE, Cumulative Index to Nursing and Allied Health Literature (CINAHL), Web of Science, and Scopus databases and will be based on the keywords described in Table 1. The search strategy will be implemented in three steps, namely: (1) an initial search limited to databases, followed by analysis of the keywords in the titles, abstracts, and index terms used to describe the articles in each of these databases; (2) a second search using all the keywords and terms indexed in all the databases included; and (3) a search for additional studies in the reference lists of the articles obtained that were not previously retrieved in the literature search in the databases. The full search strategy for each database is detailed in Appendix A.

### 2.5. Study Screening and Selection

After the literature search, all references will be gathered in the bibliographic reference manager Mendeley (Elsevier, Alpharetta, GA, USA), and duplicated references will be removed. All results will be uploaded to the Rayyan platform, a software tool allowing reviewers to collaborate on screening studies [67]. Titles and abstracts will be screened by two independent reviewers (P.C. and P.L.) considering the review’s inclusion and exclusion criteria. Reviewers will rate each study as “include”, “exclude”, or “unclear”, meaning not enough information. Studies identified as “unclear” will be retrieved in full text for eligibility assessment. The same reviewers will independently analyze the full-text articles to identify those that meet the inclusion criteria and those that do not. Studies that do not meet the inclusion criteria will be excluded, and the reasons will be reported. Lack of agreement between reviewers and doubts about the eligibility of a study will be resolved through discussion or on the basis of a third-party reviewer (F.G.). The results of the screening, search, and selection of studies will be reported using the flowchart of the Preferred Reporting Items for Systematic Reviews and Meta-Analyses (PRISMA) [62]. In this flowchart, we will present the number and reasons for the exclusion of articles.

### 2.6. Assessment of Methodological Quality

Two researchers will independently assess the methodological quality of the selected studies (P.C. and P.L.). To perform this assessment, we will use the Critical Appraisal Skills Programme Appraisal Tool for Qualitative Research (CASM) Tool [68]. This tool allows researchers to determine the risk of bias for each article, ensuring that the themes identified are from valid and reliable sources. This tool has ten questions addressing three main areas of qualitative studies: (1) internal validity; (2) results; and (3) external validity. Each question has three answer options: “yes”, ”no”, and “cannot tell”. Researchers will grade each study from A to D, depending on the degree to which it meets the methodological quality criteria. A third reviewer will be consulted to decide when classifications do not agree (F.G.). The results of the methodological quality assessment will be reported in a table, indicating, if applicable, the articles that were excluded and the corresponding reasons.

### 2.7. Data Extraction

The extracted data will be presented in a table with the main information from the chosen studies. This table will include two sections: one to register the characteristics of the study and the other to register the extracted information that answers the review question. The first section will include: (1) information about the authors, year of publication, and country; (2) the objective of the study; (3) participants and the clinical setting of the study; (4) the research design; and (5) the data collection method. To extract these data, we will use the section on methods and materials of the studies as an information source. The second section will only include a description of the influencing factors. These data will be extracted from the results and conclusion sections of each study. To visually display the data, all selected studies will be uploaded to NVivo (QRS International, Burlington, MA, USA). This software facilitates researchers’ ability to systematically and rigorously perform data synthesis [69].

### 2.8. Data Synthesis

The thematic synthesis will be based on the methodology described by Thomas and Harden [60]. For this purpose, we will analyze the results section of each study, including, in the first stage, the line-by-line coding of the results directly related to the nurses’ perceptions of the factors influencing the NBH. Subsequently, those codes will be compared with each other, identifying similarities and differences that make it possible for their combination in a two-level hierarchical tree structure. Consequently, new codes will be created to cluster the initial codes. Based on inductive reasoning, analytical themes will be generated from the previous codes. To analyze the experiences of nurses within the selected studies, researchers will adopt a critical realism perspective. Critical realism is focused on a reality stratified into three domains: empirical, actual, and real [69]. This philosophical perspective is useful not only for understanding how and why NBH occurs but also for comprehending the context in which it occurs.

## 3. Results

Extracted data will be reviewed, categorized, and synthesized in table and narrative formats in order to answer the review question. These data will be organized in three subsections: (1) factors related to patients; (2) factors related to nurses; and (3) factors related to clinical care. In these three subsections, we will describe the generated analytical themes. This description will be accompanied by citations to illustrate whether they are from the studies’ participants or their authors.

## 4. Discussion

The results will be discussed considering the current literature. Based on the richness of the data, the authors will seek to systematize the review’s outputs into a conceptual framework guiding nurse managers’ actions to decrease the nurses’ inconsistent practice. The limitations and implications of the study for NBH practices and future research will also be discussed.

## 5. Conclusions

The findings of this review may provide a robust summary of the factors that influence nurses’ practice during the NBH and will offer important theoretical support for advanced nursing practice, research, and nursing management.

## Figures and Tables

**Table 1:** PEOS framework and keywords.

Structure	Keywords with the Boolean Operators and Truncations
P	nurs*
E	bedside handover* OR bedside handoff OR bedside clinical handover* OR bedside clinical handoff* OR bedside shift-to-shift handover* OR bedside shift-to-shift handoff* OR bedside shift report* OR change-of-shift bedside report* OR change-of-shift bedside handover* OR change-of-shift bedside handoff* OR shift report at bedside
O	attitude* OR perception* OR belief* OR experience* OR opinion* OR behaviour* OR behavior*
S	NOT review NOT quantitative

* Truncations.

## Appendix A

**Table A1:** Full search strategies for each database.

Database	Search Strategy
**MEDLINE**	((((nurs*[Title/Abstract]) AND (bedside handover*[Title/Abstract] OR bedside handoff[Title/Abstract] OR bedside clinical handover*[Title/Abstract] OR bedside clinical handoff*[Title/Abstract] OR bedside shift-to-shift handover*[Title/Abstract] OR bedside shift-to-shift handoff*[Title/Abstract] OR bedside shift report*[Title/Abstract] OR change-of-shift bedside report*[Title/Abstract] OR change-of-shift bedside handover*[Title/Abstract] OR change-of-shift bedside handoff*[Title/Abstract] OR shift report at bedside[Title/Abstract])) AND (attitude*[Title/Abstract] OR perception*[Title/Abstract] OR belief*[Title/Abstract] OR experience*[Title/Abstract] OR opinion*[Title/Abstract] OR behaviour*[Title/Abstract] OR behavior*[Title/Abstract])) NOT (review[Title/Abstract])) NOT (quantitative[Title/Abstract Filters: English
**CINAHL**	AB NUR* AND AB (bedside handover* OR bedside handoff OR bedside clinical handover* OR bedside clinical handoff* OR bedside shift-to-shift handover* OR bedside shift-to-shift handoff* OR bedside shift report* OR change-of-shift bedside report* OR change-of-shift bedside handover* OR change-of-shift bedside handoff* OR shift report at bedside) AND AB (attitude* OR perception* OR belief* OR experience* OR opinion* OR behaviour* OR behavior*) NOT AB review NOT AB quantitative
**Web of Science**	((((TI = (nurs*)) AND AB = (bedside handover* OR bedside handoff OR bedside clinical handover* OR bedside clinical handoff* OR bedside shift-to-shift handover* OR bedside shift-to-shift handoff* OR bedside shift report* OR change-of-shift bedside report* OR change-of-shift bedside handover* OR change-of-shift bedside handoff* OR shift report at bedside)) AND ALL = (attitude* OR perception* OR belief* OR experience* OR opinion* OR behaviour* OR behavior*)) NOT ALL = (review)) NOT ALL = (quantitative) and English (Languages)
**Scopus**	nurs* AND ((bedside AND handover*) OR (bedside AND handoff*) OR (bedside AND clinical AND handover*) OR (bedside AND clinical AND handoff*) OR (bedside AND shift-to-shift AND handover*) OR (bedside AND shift-to-shift AND handoff*) OR (bedside AND shift AND report*) OR (change-of-shift AND bedside AND report*) OR (change-of-shift AND bedside AND handover*) OR (change-of-shift AND bedside AND handoff*) OR (shift AND report AND at AND bedside)) AND (attitude* OR perception* OR belief* OR experience* OR opinion* OR behaviour* OR behavior*) AND NOT review AND NOT quantitative AND (LIMIT-TO (SUBJAREA, “NURS”)) AND (LIMIT-TO(LANGUAGE, “English”))
